# Predictive factors for successful INTELLiVENT-ASV® use: a retrospective observational study

**DOI:** 10.1186/s12871-020-01014-w

**Published:** 2020-04-25

**Authors:** Shinshu Katayama, Ken Tonai, Jun Shima, Kansuke Koyama, Shin Nunomiya

**Affiliations:** Division of Intensive Care, Department of Anesthesiology and Intensive Care Medicine, Jichi Medical University School of Medicine, 3311-1, Yakushiji, Shimotsuke, Tochigi, 329-0498 Japan

**Keywords:** Closed loop ventilation, INTELLiVENT-ASV®, Mechanical ventilation, APACHE II score, Post-elective surgery

## Abstract

**Background:**

INTELLiVENT-ASV**®** (I-ASV) is a closed-loop ventilation mode that automatically controls the ventilation settings. Although a number of studies have reported the usefulness of I-ASV, the clinical situations in which it may be useful have not yet been clarified. We aimed to report our initial 3 years of experience using I-ASV, particularly the clinical conditions and the technical and organizational factors associated with its use. Furthermore, we evaluated the usefulness of I-ASV and determined the predictive factors for successful management with I-ASV.

**Methods:**

This single-center, retrospective observational study included patients who were ventilated using the Hamilton G5**®** ventilator (Hamilton Medical AG, Rhäzüns, Switzerland) from January 2016 to December 2018. The patients were categorized into the “I-ASV success” group and “I-ASV failure” group (those receiving mechanical ventilation with I-ASV along with any other mode). Multivariate analysis was performed to identify factors associated with successful I-ASV management.

**Results:**

Of the 189 patients, 135 (71.4%) were categorized into the I-ASV success group. In the I-ASV success group, the reasons for ICU admission included post-elective surgery (94.1%), post-emergent surgery (81.5%), and other medical reasons (55.6%). I-ASV failure was associated with a low P/F ratio (278 vs. 167, *P* = 0.0003) and high Acute Physiology and Chronic Health Evaluation (APACHE) II score (21 vs. 26, *P* < 0.0001). The main reasons for not using I-ASV included strong inspiratory effort and asynchrony. The APACHE II score was an independent predictive factor for successful management with I-ASV, with an odds ratio of 0.92 (95% confidential interval 0.87–0.96, *P* = 0.0006). The area under the receiver operating curve for the APACHE II score was 0.722 (cut-off: 24).

**Conclusions:**

In this study, we found that 71.4% of the fully mechanically ventilated patients could be managed successfully with I-ASV. The APACHE II score was an independent factor that could help predict the successful management of I-ASV. To improve I-ASV management, it is necessary to focus on patient-ventilator interactions.

## Background

INTELLiVENT-ASV**®** (I-ASV) is a closed-loop ventilation mode that automatically adjusts the ventilator settings of adaptive support ventilation (ASV). It automatically controls the fraction of inspiratory oxygen (F_I_O_2_), percentage minute ventilation (%MV), and positive end-expiratory pressure (PEEP) by using end-tidal carbon dioxide tension (E_T_CO_2_), respiratory rate, and arterial oxygen saturation of pulse oximetry (SpO_2_) to keep the patient’s lung ventilated safely. I-ASV can be managed using only one mode during the entire mechanical ventilation period, without pressure-controlled ventilation (PCV) or pressure support ventilation (PSV). The ASV algorithm is based on the minimal work and force of breathing [1], which is related with the minimal inspiratory pressure and tidal volume. Among the oxygen parameters, F_I_O_2_ and PEEP are adjusted automatically to reach a target SpO_2_ and prevent over-oxygenation. Although several studies have reported the usefulness of I-ASV [2–7], the clinical situations in which it should be used have not yet been clarified. Moreover, there are few experienced facilities where I-ASV can be used, and therefore, its usage status and efficacy have not been reported.

In the current study, we aimed to evaluate the factors which affected to I-ASV success from our initial 3 years of experience with this mode, not only the factors of clinical conditions but also the technical and organizational factors associated with its use. Moreover, we aimed to determine the associated factors in patients who could be ventilated successfully only with I-ASV.

## Methods

### Study design and setting

This was a single-center, retrospective, observational study conducted in the general intensive care unit (ICU) of a University Hospital (Tochigi, Japan). Patients who received mechanical ventilation (MV) using a Hamilton G5**®** ventilator (Hamilton Medical AG, Rhäzüns, Switzerland) (G5) in the ICU from January 2016 to December 2018 were included in this study. Clinical decisions to change the ventilation mode were made at the discretion of the attending ICU physicians. The study protocol was approved by the Institutional Research Ethics Committee of the Jichi Medical University Hospital (A19–045). Informed consent was waived due to the retrospective nature of the study.

### Participants

Patients were eligible for enrolment if they were (a) 20 years of age or older and (b) ventilated using the G5 during their ICU stay. Patients who were younger than 20 years of age or who were ventilated using other ventilators when starting MV were excluded from the study.

The baseline characteristics of the patients, including age, sex, height, body weight, body mass index (BMI), presence of sepsis, type of ICU admission, and reasons for MV were collected from the electronic medical records. The PaO_2_/F_I_O_2_ (P/F) ratio on day 1, the use of vasopressors on day 1, the use of analgesics (fentanyl, and/or morphine) and neuromuscular blockade, the use of continuous renal replacement therapy (CRRT), and the use of extracorporeal membrane oxygenation (ECMO) were also determined. The medical history, including the presence of end-stage renal function on hemodialysis and the hemi-lung status, was evaluated. Regarding ventilatory parameters, the type of ventilation mode and the duration of MV were evaluated. The reason for changing from I-ASV to a different ventilator mode was determined from the electronic medical records. The Acute Physiology and Chronic Health Evaluation II (APACHE II) score [8] and the Sequential Organ Failure Assessment (SOFA) score [9] on day 1 were used to assess the severity of each patient’s illness. The mortality rates during the ICU stay were also evaluated. In our ICU, we have more than 10 years of experience using a sedation analgesia protocol for ventilated patients. In brief, 10-30 μg/h of fentanyl is the first choice analgesics to maintain patients at Numeric Rating Scale (NRS) of 0–2. In case of severe inspiratory drive, we use morphine 1-3 mg/h instead of fentanyl. In regard to sedation, our main goal of level of sedation in mechanically ventilated patients during daytime is Richmond Agitation-Sedation Scale (RASS) of − 1 ~ 0 and − 2 ~ − 3 during night time. The choice of sedatives and analgesics and the use of rescue haloperidol depend on each attendant physician’s decision. Neuromuscular blockage is allowed to use within 48 h only for patients who present P/F ratio < 150, presence of severe respiratory effort or severe asynchronies. In this study, we evaluated the proportion of I-ASV success as the primary endpoint.

In our ICU, I-ASV is the first-choice mode of ventilation for all patients connected to a Hamilton G5 ventilator. We generally use automatic control of %MV for all patients, but the use of automatic control of F_I_O_2_ or PEEP depends on each attendant physician’s decision. Cases mainly ventilated in I-ASV mode (apart from brief phases) were considered I-ASV successes. Brief phases of I-ASV switch-off were attributed to (a) PCV immediately after tracheal intubation, (b) PSV for a manual spontaneous breathing trial (SBT) although I-ASV succeeded SBT automatically, and (c) a lack of medical staff experience with I-ASV (e.g., the activation of SpO_2_ and E_T_CO_2_ sensors on the G5). In the absence of physicians experienced with I-ASV, our clinical protocols allowed physicians without these specific skills to switch off this advanced mode and use a conventional mode. In such cases, I-ASV was re-started as soon as possible. Brief phases of I-ASV switch-off were defined as periods of < 24 h in a conventional mode before switching back to I-ASV. Patients who did not meet that criterion were classified into I-ASV failure group, which also included patients who did not receive I-ASV during MV.

### Statistical analysis

All analyses were performed using JMP 14 pro (SAS Institute Inc., Cary, NC, USA). Data are presented as medians and interquartile ranges (25th–75th percentiles) or as percentages. Categorical variables were compared between groups using Fisher’s exact test, Student’s t-test, and the Pearson chi-square test as appropriate. The subgroup analyses were performed according to the type of ICU admission (post-elective surgery, post-emergency surgery, and medical reasons) and degree of hypoxemia (P/F < 100, 100–200, 200–300, and ≥ 300). To evaluate the factors associated with successful management using I-ASV, a logistic regression analysis was performed to determine the non-adjusted and adjusted odds ratios (ORs) using model 1 (age, sex, BMI, presence of sepsis, end-stage renal function on hemodialysis, P/F ratio, and APACHE II score) and model 2 (P/F ratio and APACHE II score). The variables of this model were selected manually which could clinically affect I-ASV success. The number of included variables in multivariate analysis were limited because of low sample size. A forward stepwise elimination process was used to remove non-significant variables from the model. Using the area under the receiver operating characteristic curve (AUROC), we assessed the ability of each independent factor to predict successful ventilation with I-ASV [10]. *P*-values < 0.05 were considered statistically significant.

## Results

### Enrolment and baseline characteristics

**Fig. 1:**
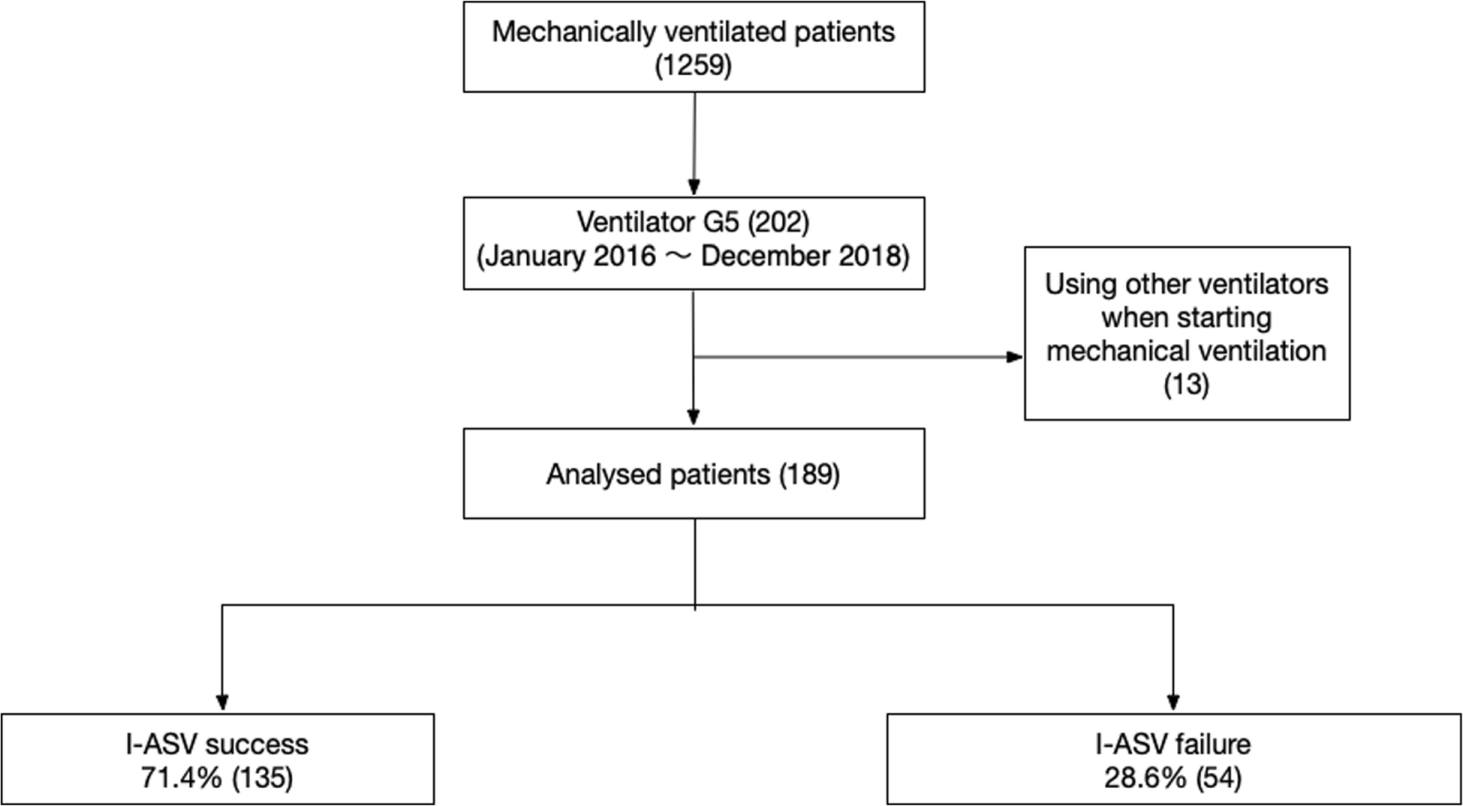
Flow chart showing the classification of the study patients

A total of 202 patients who received MV with G5 were enrolled in the study (Fig. 1). Thirteen patients had been ventilated with other ventilators when starting MV. One hundred and thirty-five patients (71.4%) were classified into the I-ASV success group, and 54 (28.6%) were categorized into the I-ASV failure group. In the I-ASV success group, some patients had briefly received another ventilation mode for reasons such as PCV immediately after tracheal intubation (*n* = 5, 3.7%), PSV for manual SBT although I-ASV revealed SBT success (*n* = 7, 5.2%), and a lack of medical staff experience with I-ASV (*n* = 9, 6.7%). Automatic control of each %MV, F_I_O_2_, and PEEP were used in 100, 85.6, and 14.4% of patients, respectively.

Table 1 compares the characteristics of patients in the I-ASV success and I-ASV failure groups. I-ASV failure was associated with a high APACHE II score (21 vs. 26, *P* < 0.0001), SOFA score (day 1) (8 vs. 11, *P* < 0.0001), CRRT (10.4% vs. 44.4%, *P* < 0.0001), use of vasopressors (day 1) (52.6% vs. 70.4%, *P* = 0.034), use of morphine (5.2% vs. 27.8%, *P* < 0.0001), use of neuromuscular blockade (4.4% vs. 25.9%, *P* < 0.0001), type of ICU admission (*P* < 0.0001) and low P/F ratio (278 vs. 167, *P* = 0.0003). Additionally, the duration of MV (5 vs. 10 days, *P* < 0.0001), length of ICU stay (6 vs. 11 days, *P* < 0.0001), MV after ICU (i.e.,, continued MV after ICU discharge, such as in the general ward or at a transferred hospital; 9.6% vs. 22.2%, *P* = 0.031), and ICU mortality (1.5% vs. 13.0%, *P* = 0.003) were all significantly lower in the I-ASV success group (Table 1).

**Table 1:** Patient characteristics

	All patients	I-ASV success	I-ASV failure	*P*-value
	(*n* = 189)	(*n* = 135)	(*n* = 54)	
Age, years	65 (52–74)	65 (50–74)	65 (54–74)	0.555
Sex, male	52.9%	50.4%	59.3%	0.333
Height, cm	160 (153–167)	159 (153–165)	162 (153–169)	0.160
Weight, kg	58 (50–67)	58 (51–67)	59 (50–68)	0.448
BMI, kg/m^2^	22.6 (20.7–25.8)	22.6 (20.8–25.3)	22.5 (19.9–27.3)	0.798
APACHE II	23 (17–27)	21 (15–26)	26 (21–34)	<.0001
Presence of sepsis	52.9%	48.9%	61.1%	0.148
CKD on HD	6.9%	5.9%	9.3%	0.525
CRRT	20.1%	10.4%	44.4%	<.0001
SOFA (day1)	9 (6–12)	8 (6–11)	11 (8–15)	<.0001
Use of vasopressors (day1)	57.7%	52.6% (71)	70.4% (38)	0.034
Reasons for admission				<.0001
Elective surgery		94.1% (32)	5.9% (2)	
Emergent surgery		81.5% (53)	18.5% (12)	
Medical reasons		55.6% (50)	44.4% (40)	
Sedatives and analgesics				
Fentanyl		100%	100%	–
Morphine		5.2%	27.8%	<.0001
Neuromuscular blockade		4.4%	25.9%	<.0001
Reasons for ventilation				<.0001
Respiratory failure		15.6% (21)	50.0% (27)	
Hemodynamic instability		8.9% (12)	13.0% (7)	
Central nervous dysfunction		11.1% (15)	7.4% (4)	
Sepsis		33.3% (45)	16.7% (9)	
Highly invasive surgery		11.9% (16)	1.9% (1)	
Flap surgery		9.6% (13)	0% (0)	
Post resuscitation		3.0% (4)	3.7% (2)	
Trauma		3.0% (4)	3.7% (2)	
Other		3.7% (5)	3.7% (2)	
P/F ratio	252 (174–334)	278 (206–366)	167 (98–246)	0.0003
P/F ratio classification				<.0001
≥ 300		89.4% (84)	10.6% (10)	
≤ 200 to < 300		71.4% (25)	28.6% (10)	
≤ 100 to < 200		47.5% (19)	52.5% (21)	
< 100		35.0% (7)	65.0% (13)	
MV duration, days	6 (4–10)	5 (3–8)	10 (7–16)	<.0001
MV after ICU	13.2%	9.6%	22.2%	0.031
ICU mortality	4.8%	1.5%	13.0%	0.003
ICU duration, days	7 (5–11)	6 (4–9)	11 (8–16)	<.0001

Acute Physiology and Chronic Health Evaluation II, APACHE II; body mass index, BMI; chronic kidney disease on hemodialysis, CKD on HD; continuous renal replacement therapy, CRRT; extracorporeal membrane oxygenation, ECMO; intensive care unit. ICU; INTELLiVENT-ASV**®**, I-ASV; mechanical ventilation, MV; PaO_2_/F_I_O_2_ ratio, P/F ratio, sequential organ failure assessment score; SOFA score.

### Reasons for admission and MV, and severity of hypoxemia associated with I-ASV success group

The reasons for ICU admission and MV, and the severity of hypoxemia were evaluated in I-ASV success group. Notably, I-ASV success group included 94% of the patients admitted for post-elective surgery, 81.5% of those admitted for post-emergency surgery, and 55.6% of those with other medical reasons. As for the reasons for ventilation, I-ASV success was associated with central nervous dysfunction, sepsis, highly invasive surgery, and flap surgery. Regarding the severity of hypoxemia, 89.4, 71.4, 47.5, and 35.0% of patients with a P/F ratio ≥ 300, 200–300, 100–200 and, < 100, respectively, were classified into I-ASV success group (Table 1).

### Annual trends regarding successful I-ASV

The number of patients who were ventilated using G5 increased annually. Among these patients, I-ASV was successful in 69.0% of patients in 2016, 72.1% in 2017, and 71.2% in 2018. Compared to patients in I-ASV failure group, those in I-ASV success group have lower APACHE II scores [19 vs. 27 in 2016 (*P* = 0.080), 17 vs. 23 in 2017 (*P* = 0.058), and 23 vs. 30 in 2018 (*P* < 0.0001)]. Based on the reasons for admission in 2018, 100% of those admitted for post-elective surgery, 90.6% of those admitted for emergent surgery, and 54.0% of those with other medical reasons (*P* = 0.0002) were classified into I-ASV success group. Regarding the severity of hypoxemia in 2018, 92.3, 75.0, 52.6, and 35.7% of patients with a P/F ratio of ≥300, 200–300, 100–200, and < 100, respectively (*P* = 0.0001), were classified into I-ASV success group (Table 2).

**Table 2:** Annual trends associated with successful I-ASV

Year	2016	2017	2018
Number of included patients	*n* = 29	*n* = 68	*n* = 92
I-ASV success, (n)	69.0% (20)	72.1% (49)	71.7% (66)
APACHE II	19 (17–27)	17 (15–25)	23 (18–27)
SOFA (day1)	8 (5–10)	7 (6–10)	10 (7–13)
Use of vasopressor (day1)	50.0%	46.9%	57.6%
P/F ratio	281 (227–365)	290 (234–391)	256 (178–337)
Presence of sepsis	63.6%	67.7%	66.7%
CKD on HD	50.0%	66.7%	75.0%
Trauma	0.0%	66.7%	100%
Reasons for admission			
Elective surgery	85.7%	94.1%	100.0%
Emergent surgery	69.2%	75.0%	90.6%
Medical reasons	55.6%	58.1%	54.0%
P/F ratio classification			
≥ 300	82.4%	89.5%	92.3%
≤ 200 to < 300	50.0%	72.7%	75.0%
≤ 100 to < 200	60.0%	37.5%	52.6%
< 100	33.3%	33.3%	35.7%

Acute Physiology and Chronic Health Evaluation II, APACHE II; chronic kidney disease on hemodialysis, CKD on HD; INTELLiVENT-ASV**®**, I-ASV; PaO_2_/F_I_O_2_ ratio, P/F ratio, sequential organ failure assessment score; SOFA score.

### Reasons for choosing other modes of ventilation

**Fig. 2:**
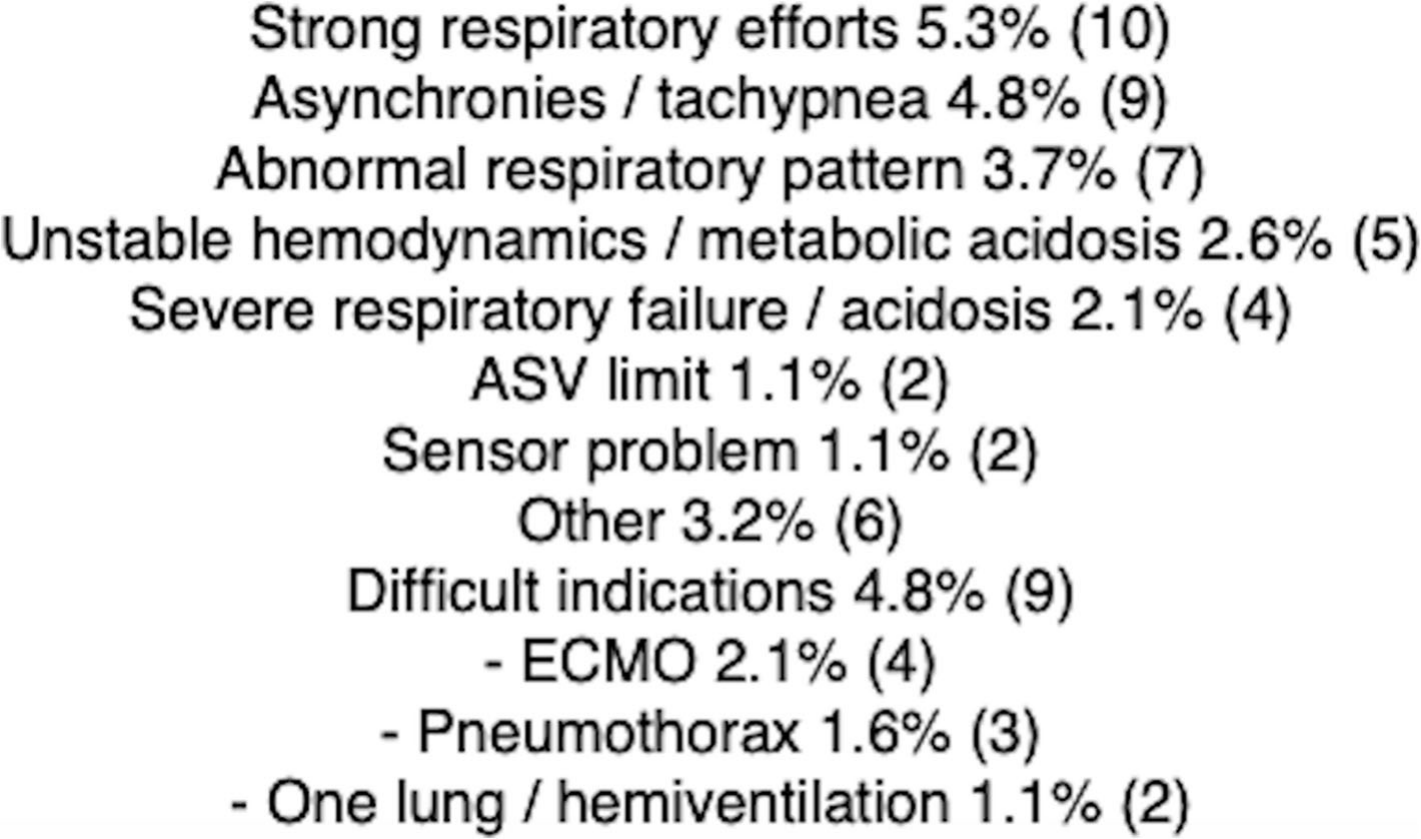
The reasons for choosing other modes of ventilation. ASV, adaptive support ventilation; extracorporeal membrane oxygenation, ECMO

Figure 2 summarizes the reasons for choosing other modes of ventilation in I-ASV failure group. The main reasons included patients’ strong respiratory efforts (*n* = 10, 5.3%); asynchrony/tachypnoea (*n* = 9, 4.8%); abnormal respiratory patterns, including Cheyne-Stokes respiration and an opioid-induced respiratory pattern (*n* = 7, 3.7%); unstable hemodynamic/metabolic acidosis (*n* = 5, 2.6%); severe respiratory failure/acidosis (*n* = 4, 2.1%); limitation of the ASV setting (*n* = 2, 1.1%); sensor problems (n = 2, 1.1%); other reasons (*n* = 6, 3.2%); difficult indications (*n* = 9, 4.8%), such as ECMO (*n* = 4, 2.1%), pneumothorax (*n* = 3, 1.6%), one lung and hemiventilation (*n* = 2, 1.1%).

### Multivariate analysis, ORs, and AUROC predictive of successful I-ASV

**Fig. 3:**
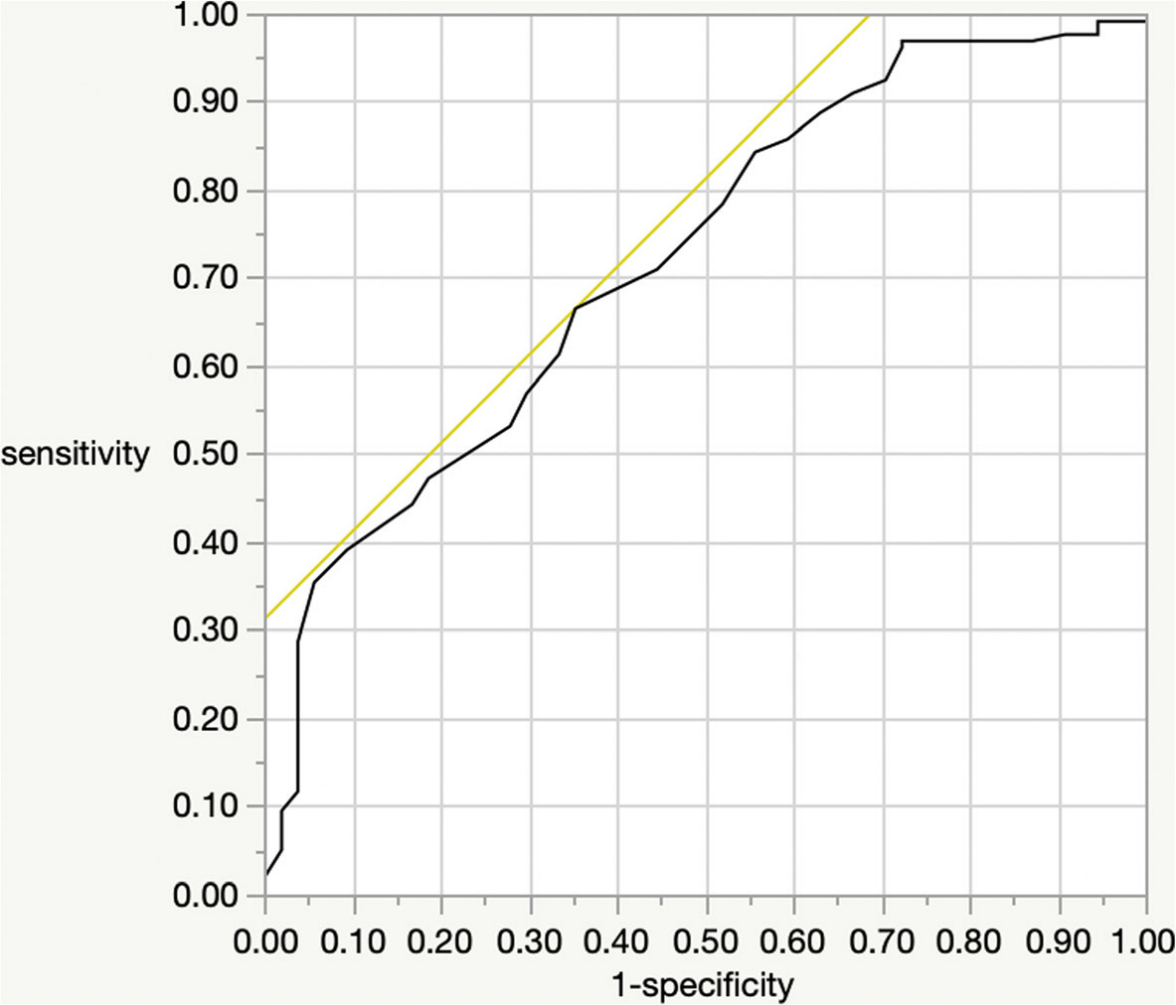
The area under the receiver operative curve compared with the APACHE II

Because of the limitation of number of studied patients (*n* = 54) in I-ASV failure group, we decided to conduct the multivariate analysis up to seven parameters which could affect the predictability of successful I-ASV (model 1). Among the seven evaluated parameters, the APACHE II score (OR: 0.92; 95% CI: 0.87–0.96; *P* = 0.0006) was found to be an independent predictor of successful I-ASV. Also, we conducted the multivariate analysis with only significant variables including APACHE II and P/F ratio (model 2). As result, the APACHE II score was an independent predictor of successful I-ASV (OR: 0.92; 95%CI: 0.88–0.96; *P* = 0.0005). The AUROC for the APACHE II score was 0.722 (0.637–0.794, cut-off: 24 [sensitivity: 0.67, specificity: 0.65]). Although stepwise regression analysis revealed P/F ratio as not an independent factor of successful I-ASV, the AUROC was 0.736 (0.644–0.812, cut-off 187 [sensitivity 0.81 specificity 0.61]). (Table 3, Fig. 3).

**Table 3:** Multivariate analysis for predicting successful ventilation with I-ASV

	Odds ratio (95% CI)	*P*-value	Odds ratio (95% CI)	*P*-value	Odds ratio (95% CI)	*P*-value
	(Non-adjusted)		(adjusted: model 1)		(adjusted: model 2)	
Age	0.99 (0.97–1.01)	0.550	1.01 (0.98–1.03)	0.673		
Sex, male	1.43 (0.76–2.74)	0.268	1.39 (0.69–2.80)	0.354		
BMI	0.99 (0.93–1.06)	0.798	0.99 (0.93–1.07)	0.959		
Presence of sepsis	1.64 (0.87–3.16)	0.127	0.86 (0.40–1.85)	0.697		
CKD on HD	1.62 (0.47–5.10)	0.426	1.03 (0.29–3.75)	0.960		
P/F ratio	1.005 (1.002–1.008)	<.0001	1.003 (1.000–1.006)	0.052	1.003 (1.000–1.006)	0.042
APACHE II	0.91 (0.87–0.95)	<.0001	0.92 (0.87–0.96)	0.0006	0.92 (0.88–0.96)	0.0005

Acute Physiology and Chronic Health Evaluation II, APACHE II; body mass index, BMI; confidential interval, CI; chronic kidney disease on hemodialysis, CKD on HD; INTELLiVENT-ASV**®**, I-ASV; PaO_2_/F_I_O_2_ ratio, P/F ratio.

## Discussion

We found that 71.4% of the evaluated patients were ventilated in I-ASV mode throughout their ICU stay. The I-ASV success group included more than 90% of the patients undergoing post-elective surgery but only 55.6% of those with other medical reasons. I-ASV failure was associated with a low P/F ratio and high APACHE II score. The main reasons cited for switching to other modes of ventilation were asynchrony and a strong patient inspiratory effort. Moreover, we identified the APACHE II score as a significant independent predictor of successful ventilatory management using I-ASV.

Our findings also demonstrated that in cases with few or no lung problems, ventilatory management almost exclusively with I-ASV was possible. Several studies have demonstrated the usefulness of I-ASV. Arnal et al. [2] reported that compared to ASV, I-ASV is safer and can provide ventilation with a lower pressure, volume, and F_I_O_2_ in passive patients with acute respiratory failure. In addition, Lellouche et al. [5] reported the safety of I-ASV for maintaining an optimal range of respiration in post-cardiac surgery patients. A randomized controlled trial that aimed to compare the efficacy of I-ASV with conventional ventilation methods in critically ill patients [7] found I-ASV to be a safe and effective option in terms of tidal volume, respiratory rate, SpO_2_, and E_T_CO_2_. However, these studies were performed for short durations and did not evaluate the long-term success rates of I-ASV. Therefore, our study is the first to evaluate the long-term success of I-ASV in a real clinical situation.

In the current study, the APACHE II score was an independent predictive factor for successful I-ASV. In addition, the cut-off value for the APACHE II score was 24. Along with the finding that strong respiratory drive was one of the reasons for discontinuation of I-ASV, high APACHE II scores may be related to strong respiratory drive and I-ASV failure. In line with the severity of illness or organ dysfunction, P/F ratio is also considered as the indicator of acute respiratory failure and/or acute respiratory distress syndrome (ARDS). Athough not statistically significant in multivariate analysis, P/F ratio was indeed associated with I-ASV success (Table 1). Moreover, the AUROC of P/F ratio < 186 was 0.736, and this was even slightly higher than the AUROC of APACHE II. Relatively small number of patients in this study might influence these results. However, these results also suggest that in cases of moderate to severe ARDS or severe multi-organ dysfunction, management by I-ASV alone may be quite difficult.

In this study, we compared the annual trends associated with successful I-ASV. Initially in the first year, we actually used I-ASV mainly for relatively low APACHE II score, such as post-operative patients. With the accumulation of the experience of using I-ASV, we gradually used I-ASV for more severe patients like ARDS patients. Considering the learning curve to learn how I-ASV work, and even if it appears simple at first sight, we think that medical stuff knowledge and experience with I-ASV are the key to success of the management with this mode. In addition, when compared to other conventional ventilation methods, I-ASV is expected to reduce the burdens on medical staff members (e.g., physicians and nurses) because of the automation. Although there is little evidence regarding the reduction in medical staff workloads, I-ASV might be a better choice for the majority of mechanically ventilated patients.

I-ASV may be difficult to manage in some cases because of the strong respiratory effort required in patients with moderate to severe hypoxemia and respiratory/metabolic acidosis. A strong respiratory effort frequently requires high minute ventilation. As I-ASV has a setting limitation of 200% of 100 mL/predictable body weight, ventilated patients who require high minute ventilation might find I-ASV to be challenging. Moreover, ventilation with I-ASV during a high inspiratory flow rate becomes difficult due to asynchrony. In such cases, it is important to switch to an appropriate ventilation mode rather than continue with I-ASV. In this study, there were a few cases in which the trigger for spontaneous inspiration was suppressed by changing to PCV. Furthermore, in such situations, the use of narcotics such as morphine, sedatives, or muscle relaxants should be considered.

Our study had several limitations. First, this was a single-center, retrospective, observational study. The number of the patients were relatively small. These may limit the power of the study. Further studies are warranted to validate our findings. Second, there was a selection bias due to the specific choice of G5. In our ICU, G5 is preferred in cases of hypoxemia and severe organ dysfunction rather than in cases involving post-elective surgery, and this preference could have influenced the success rate of I-ASV. If the number of post-elective surgery patients exceeded the number of patients with hypoxemia and severe organ dysfunction, it could have increased the success rate of I-ASV. Third, we did not correct ventilatory parameters such as the tidal volume, driving pressure, and PEEP. Therefore, we are unable to conclude whether I-ASV was superior to other conventional ventilator modes in this study. We are currently conducting a prospective study (UMIN000034417) to evaluate the usefulness of I-ASV and mechanical power compared to other conventional ventilation modes. Fourth, we did not record the types of asynchronies in detail. Hence, further studies are required to evaluate the types of asynchronies that increase the difficulty of respiratory distress management with I-ASV. Fifth, we did not have objective parameters for high respiratory efforts, like esophageal pressure. Sixth, we did not collect the type of sedative drugs and doses. Because the optimal suppression of the respiratory drive with sedatives may directly relates to the success of I-ASV, and because asynchronies, which we found in this study associated with I-ASV success, can occur in either the situation of over-sedation or under-sedation. Further studies are needed to evaluate about this issue. Finally, since the proficiency of medical staff with I-ASV mode varies, our results might differ from those of other high-volume centers. However, this study included the first 3 years of experience I-ASV in our ICU, and therefore our results may be useful with respect to the initial use of I-ASV.

Despite these possible limitations, I-ASV is useful, especially for post-elective surgery patients and patients who do not have severe hypoxemia or severe organ dysfunction. Accordingly, it is important to select patients carefully for MV with I-ASV.

## Conclusions

In this study, 71.4% of the patients could be ventilated with I-ASV, which included more than 90% of patients with post-elective surgery, but only 55.6% of those admitted for other medical reasons. I-ASV failure was associated with a low P/F ratio and high APACHE II score. Moreover, the APACHE II score was an independent factor predictive of successful management with I-ASV. The main reasons for an inability to ventilate exclusively with I-ASV included a strong inspiratory effort and asynchronies. A focus on patient-ventilator interactions is necessary to improve I-ASV management.

## Data Availability

The datasets used and/or analyzed during the current study are available from the corresponding author on reasonable request.

## Abbreviations

APACHE: Acute physiology and chronic health evaluation
ASV: Adaptive support ventilation,
AUROC: Area under the receiver operating characteristic curve
BMI: Body mass index
CKD on HD: Chronic kidney disease on hemodialysis
CRRT: Continuous renal replacement therapy
ECMO: Extracorporeal membrane oxygenation
E_T_CO_2_: End-tidal carbon dioxide tension
I-ASV: INTELLiVENT-ASV®
ICU: Intensive care unit
MV: Mechanical ventilation
%MV: Percent minute volume
NRS: Numeric Rating Scale
PCV: Pressure controlled ventilation
P/F ratio: PaO_2_/F_I_O_2_ ratio
PSV: Pressure support ventilation
RASS: Richmond Agitation-Sedation Scale
SOFA score: Sequential Organ Failure Assessment score.
